# *Notes from the Field*: Assessing Rabies Risk After a Mass Bat Exposure at a Research Facility in a National Park — Wyoming, 2017

**DOI:** 10.15585/mmwr.mm6710a7

**Published:** 2018-03-16

**Authors:** Andrea Cote, Sarah Anne J. Guagliardo, Cuc H. Tran, Maria A. Said, Veronica Pickens, Karl Musgrave, Ryan Wallace

**Affiliations:** ^1^Epidemic Intelligence Service, CDC; ^2^Wyoming Department of Health; ^3^Poxvirus and Rabies Branch, Division of High-Consequence Pathogens and Pathology, CDC; ^4^Office of Public Health, National Park Service, Washington, D.C; ^5^Epidemiology Elective Program, CDC.

On August 2, 2017, the Wyoming Department of Health (WDH) was notified by local public health nursing of a group of 20 persons who had slept in a national park research facility and reported contact with bats and bat excrement. Four of the 20 persons had already received rabies postexposure prophylaxis (PEP)* when WDH notified the National Park Service (NPS) and requested assistance from CDC for a mass bat exposure investigation of the remaining 16 persons. Rabies is a fatal, viral zoonotic disease causing an estimated 59,000 human deaths annually worldwide. Transmission from animals to humans mainly occurs through bites; however, scratches or mucous membrane contact with saliva also present transmission risks (*1*–*3*). Although human rabies in the United States is rare, most human cases result from bat exposures; 75% of infected patients become ill within 3 months of exposure (*3*). Bat infestation of human habitations increases the risk for bat contact. Infestations can expose numerous persons to rabies and are referred to as mass bat exposures.

Review of facility records identified 172 persons from 11 research groups who had slept at the research facility, with 73% of persons sleeping in one of two buildings possibly infested with bats since both buildings opened for the summer season on May 19, 2017, and closed August 2, 2017, to overnight guests. The facility director provided investigators with contact information for group leaders, who then provided contact information for potentially exposed persons. Persons resided in 29 states, the District of Columbia, one U.S. territory, and four non-U.S. residents were from four countries. Investigators from WDH, CDC, NPS, and public health professionals in other local, state, and international jurisdictions attempted to contact all potentially exposed persons by telephone, e-mail, and through social media. All persons who completed a risk assessment were contacted 1–2 weeks later to complete a follow-up assessment regarding receipt of PEP and to answer additional questions. Rabies risk assessments and follow-up assessments were conducted by telephone and e-mail.

A risk assessment tool adapted from a previous mass bat exposure investigation (*4*) was used to determine each person’s risk for rabies virus exposure. The assessment was modified with additional questions to create three risk categories based on bat contact, sightings, and whether the bedroom door was open or closed while the person was sleeping. Persons were categorized as having no risk (no direct bat contact, no bats observed, and door closed while sleeping), low risk (no direct bat contact, no bats observed, and door open while sleeping), or high risk for bat exposure (direct contact with a bat or lack of knowledge of possible bat contact while sleeping because of medications, deep sleep, or alcohol consumption).

By February 8, 2018, risk assessments had been completed for 165 (95.9%) of 172 potentially exposed U.S. residents, with the remaining persons considered lost to follow-up. Among those assessed, 123 (74.5%) persons were classified as having no exposure risk, 21 (12.7%) a low exposure risk, and 21 (12.7%) a high exposure risk. Although all persons were encouraged to consult with a health care provider if they had concerns about exposure, persons classified as having a high exposure risk were counseled regarding potential rabies virus exposure and strongly encouraged to receive PEP. All information collected from the risk assessments was shared with the appropriate public health officials. All 165 U.S. residents who stayed at the research facility and completed a risk assessment were contacted for a follow-up assessment; 79 (47.9%) completed the follow-up assessment. Among these persons, 21 (26.6%) reported receiving PEP, including five of 56 (8.9%) with no exposure risk, seven of 14 (50%) with low exposure risk, and nine of nine (100%) with high exposure risk. It is possible, however, that additional persons declining participation in the follow-up assessment might have received PEP.

As one of the largest documented mass bat exposures in U.S. history, this investigation required extensive coordination among local, state, and federal agencies, in addition to foreign governments. Public health responses to mass bat exposures vary by jurisdiction, but all work to ensure risk assessments are performed to ascertain possible rabies virus exposures that might require PEP, while also ensuring that nonexposed persons do not undergo unnecessary and costly treatment (*5*). The immediate public health response to this situation was to close the buildings to overnight guests to prevent potential rabies virus exposure to additional persons. The research facility director coordinated with a bat exclusion company to remediate and exclude areas of possible bat entry. Mass bat exposures can occur in any public building, yet no formal guidance exists for PEP administration in the context of mass bat exposures (*5*). The standardized risk assessment^†^ developed for this investigation might help guide future mass bat exposure responses to identify rabies risk among persons with potential exposures, and therefore reduce unnecessary administration of PEP.

## Footnotes

* https://www.cdc.gov/mmwr/preview/mmwrhtml/rr5902a1.htm.

† The bat exposure assessment questionnaire for adults included the following 11 questions: “1. What dates did you sleep at the [Building A], if any? What dates did you sleep at the [Building B]?”; “2. Prior to going to sleep, did you check your room for bats? Did you see any bats in the room?”; “3. Did you sleep with the bedroom door closed?”; “4. Did you sleep under a bed net?”; “5. Where did you see a bat while at the [facility] or during your trip to [park/ location]? A. in a sleeping room, B. not in a sleeping room, C. outside, D. other location, E. no bat seen. Did you hear of any bats seen inside cabins or quarters other than in [Buildings A and B]? If a bat was seen, ask to describe the dates and circumstances of each sighting. If a bat was seen, was the bat (if more than one bat was seen, collect information for each incident in the space above): A. healthy, flying normally, B. injured or apparently sick, C. dead, D. unknown”; “6. What contact did you have with a bat? Specifically, any of the following: A. bitten, B. scratched, C. touched. If yes, please describe your contact (ask specifically about touching of head/mouth/teeth and whether or not gloves were worn)”; “7. Were you asleep and then awoke to find a bat in your room during your stay? Do you recall seeing a bat swoop down or make contact with any other person while they were sleeping? If yes, do you recall the names of any other people the bat may have had contact with? (List their name and contact information)”; “8. Were you on any medications during your stay, including over the counter medications that may have made you drowsy or less likely to feel contact with a bat? If yes, please list the medications”; “9. Do you have any of the following conditions that may decrease your awareness of a bat bite? A. deep sleeper or other condition that may make them less likely to awaken if bitten by a bat, B. drug or alcohol use during your stay, C. do you normally sleep with bare skin exposed (particularly arms or legs), D. other (please list)”; “10. Have you ever been vaccinated against rabies? If yes, in what year did you receive vaccination? If yes, how many doses of vaccine did you receive?”; and “11. Are you willing to speak with us at a different time regarding possible bat sightings?”
